# Adverse Childhood Experiences and Dental Care Utilization During Pregnancy: Findings from the North and South Dakota PRAMS, 2017–2021

**DOI:** 10.21203/rs.3.rs-3452502/v1

**Published:** 2023-10-19

**Authors:** Alexander Testa, Dylan B. Jackson, Allison Crawford, Rahma Mungia, Kyle T. Ganson, Jason M. Nagata

**Affiliations:** University of Texas Health Science Center at Houston; Johns Hopkins Bloomberg School of Public Health; University of Texas Health Science Center at San Antonio; University of Texas Health Science Center at San Antonio; University of Toronto; University of California, San Francisco

**Keywords:** Adverse childhood experiences, dental care utilization, pregnancy, prenatal, PRAMS

## Abstract

**Background:**

Research demonstrates adverse childhood experiences (ACEs)—i.e., experiences of abuse, neglect, and household dysfunction—adversely impact healthcare utilization over the life course. Several studies demonstrate that ACEs are related to lower dental care utilization in childhood and adolescence. However, limited research has explored the connection between ACEs and dental care utilization in adulthood, and no research has examined this relationship during pregnancy. The current study extends existing research by investigating the relationship between ACEs and dental care utilization during pregnancy.

**Data:**

Data are from the 2017–2021 Pregnancy Risk Assessment Monitoring System (PRAMS) North Dakota and South Dakota (*n* = 7,391). Multiple logistic regression is used to examine the relationship between the number of ACEs and dental care utilization.

**Findings:**

Relative to respondents with 0 ACEs, those with 4 or more ACEs were significantly less likely to report having dental care during pregnancy (OR = 0.745, 95% CI = .628, .883). By racial and ethnic background, the results showed that the significant associations are concentrated among White and Native American respondents.

**Conclusions:**

The results suggest that exposure to 4 or more ACEs is associated with a significantly lower likelihood of dental care utilization in adulthood, and this relationship is concentrated among White and Native American respondents. Further investigations are necessary to understand the mechanisms underlying the relationship between ACEs and dental care utilization and replicate the findings in other geographic contexts.

## Introduction

Adverse childhood experiences (ACEs), encompassing abuse, neglect, and household dysfunction before age 18,^1^ have been associated with various negative long-term consequences throughout the life course. For instance, prior research documents that individuals exposed to ACEs have poorer overall health^2–4^ and lower rates of preventive healthcare utilization^5^ compared to individuals without ACE exposure. Notably, the negative impacts of ACEs on health and healthcare utilization operate in a dose-response manner, with persons exposed to more ACEs, especially four or more, often enduring worse health outcomes.^2–6^ However, less is known about how ACEs—including accumulating ACE exposure—influence important pregnancy-related outcomes and behaviors,^7^ such as dental care utilization.^8^

Routine preventive dental care is a critical social determinant of oral and overall health,^9,10^ especially during pregnancy.^8^ Unfortunately, many Americans in the United States do not regularly utilize dental care, with especially stark disparities among racial and ethnic minorities.^9,11–13^ Importantly, research suggests ACEs exposure may serve as a barrier to dental care utilization among children and adolescents.^14–18^ Even so, the literature on ACEs and dental care utilization remains limited in several key ways.

First, existing research focuses primarily on the negative influence of ACEs on dental care utilization in childhood and adolescence.^14–18^ Yet, while dental care utilization in childhood and adolescence is primarily influenced by parent decision-making, there is a lack of research on the long-term effects of ACE exposure on dental care utilization in adulthood, despite ample evidence that ACEs continue to impact health, behaviors, and healthcare utilization into adulthood.^2–6^ Indeed, there have been only two studies on ACEs and dental care utilization among adults. Both studies, which employ data from the 2010 Behavior Risk Factor Surveillance System, found that compared with participants with no ACEs, participants with ACEs—particularly four or more ACEs—were significantly more likely to report not having a dental cleaning in the past year^19^ or going two or more years since last dental cleaning.^20^

Second, little is known about the long-term impacts of ACEs on dental care utilization during specific life stages, such as pregnancy. Given that ACEs can shape maternal and infant health,^7,21,22^ and dental care is crucial during pregnancy,^23^ it is important to understand how early ACE exposure may affect dental care patterns during this critical time. Moreover, while proximal stressful life events experienced among adults around pregnancy are associated with lower dental care utilization,^24^ it remains unknown if exposure to more distal stressors, such as ACEs, affects dental care utilization during pregnancy. Finally, there is a lack of knowledge about how the relationship between ACEs and prenatal dental care utilization varies across racial and ethnic groups. This is an important gap in the existing research considering the stark racial disparities in ACEs exposure,^25^ dental care utilization,^9,11–13^ and adverse pregnancy outcomes.^26^

To address these gaps, the current study uses data from the Pregnancy Risk Assessment Monitoring System (PRAMS) from 2017–2021 in two states (North Dakota and South Dakota), including questions about ACEs exposure and dental care utilization. Specifically, we seek to accomplish the following two aims: (1) assess the relationship between ACEs exposure and dental care utilization during pregnancy, and (2) explore how this relationship varies across racial and ethnic groups.

## Methods

### Data

The data used in this study were sourced from the PRAMS, an ongoing population surveillance system conducted by the Centers for Disease Control and Prevention (CDC) and state health departments in the United States. The PRAMS collects information on live births through a stratified systematic sample of birth certificate records. The data come from three main sources: (1) birth certificate records, (2) vital record systems, and (3) responses to a PRAMS survey. The survey is sent to recent mothers’ home addresses approximately 2 to 4 months after giving birth. To ensure the representativeness of the samples, survey weights are used to adjust for non-response and non-coverage, making the samples representative of live births within a specific state.^27^ The current study uses data from the North Dakota and South Dakota PRAMS from 2017–2021, which are the only states that include questions about mothers’ adverse childhood experiences and dental care utilization (*n* = 7,391).

### Dependent Variable

*Dental care* utilization is based on a survey question asking, “During your most recent pregnancy, did you have your teeth cleaned by a dentist or dental hygienist?” (1 = yes, 0 = no).^24,28,29^

### Independent Variable

ACEs were assessed through self-report by the respondents, focusing on 10 types of childhood adversity experienced before age 18, which align with the measures from the CDC-Kaiser ACE Study.^1^ Appendix A provides detailed definitions and prevalence rates for these ten items. Following the approach of previous research that used PRAMS data, the responses to the 10 ACE items were combined to create a cumulative score ranging from 0 to 10, and the total ACEs scores were then categorized into the following mutually exclusive groups: 0 ACE, 1 ACE, 2 ACEs, and 3 ACEs, and 4 or more ACEs.^7,30,31^

### Control Variables

The control variables in this study include the *mother’s age* (< 18, 18–24, 25–29, 30–34, and 35 or older), *mother’s race/ethnicity* (White, Hispanic, Black, Native American, and other race/ethnicity), *mother’s educational attainment* (less than high school, high school graduate, some college, college graduate), mother is *currently married* (yes or no), the *number of prior births* (0, 1, 2, or 3 or more) whether a mother reported being on *Medicaid* in the three months before pregnancy (yes or no), household income (≤$16,000, $16,000-$40,000, $40,001-$85,000, or >$85,000), the *number of dependents* (0, 1, 2, or 3+), the *state of residence and year of birth*

### Statistical Methods

First, we present the summary statistics of the analytic sample and the distribution of dental care utilization stratified by the number of ACEs. Bivariate differences in the relationship between ACEs exposure and dental care utilization are assessed using a chi-square (*χ*^2^). Next, the association between ACEs and the oral outcome variables are assessed using multiple logistic regression analyses, given the binary nature of the dependent variable. Finally, we then stratify the analyses by race and ethnicity and assess differences in the magnitude of the coefficients via an equality of coefficients test.^32,33^ Analyses are conducted using Stata version 17. Models adjust for survey weights and strata information to account for the complex survey design of PRAMS.

## Results

**Figure 1:**
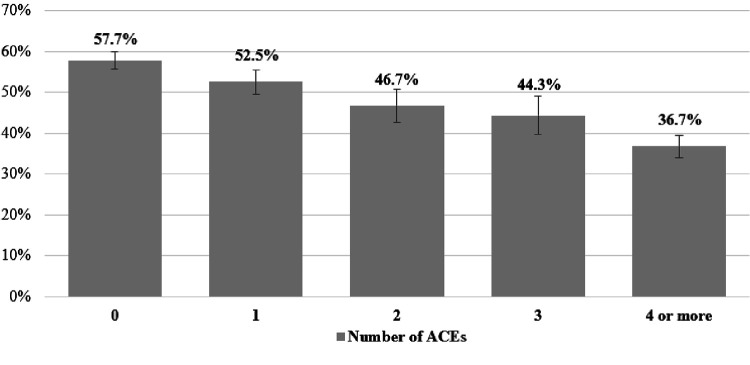
Dental Care Utilization Stratified by Number of ACEs (*N* = 7,391) *Abbreviation*: ACEs = Adverse Childhood Experiences

Table 1 presents the summary statistics of the analytic sample. Overall, 49.7% reported utilizing dental care during pregnancy, 39.1% reported no ACEs exposure, 19.7% had 1 ACE, 11.1% reported 2 ACEs, 8.1% reported 3 ACEs and 22.0% had 4 or more ACEs. Figure 1 shows that dental care utilization rates decline alongside more ACE exposure. For instance, 57.7% of respondents with 0 ACEs reported dental care utilization during pregnancy, whereas 36.4% of respondents with 4 or more ACEs reported dental care utilization (*χ*^2^ = 189.38, *p* < .001)

After accounting for control variables in Table 2, the results of the multiple logistic regression model display a similar pattern, as relative to 0 ACEs, those with 4 or more ACEs were significantly less likely to report having dental care utilization during pregnancy (OR = 0.745, 95% CI = .628, .883). The results in Table 2, stratified by race and ethnicity, show that statistically significant associations are concentrated among White and Native American respondents, especially among those with 3 or 4 or more ACEs. Equality of coefficient tests finds that there is a statistically significant difference in the coefficient of 4 or more ACEs between White and Hispanic respondents (z-score = −2.625, *p* = .009), between Native American and Hispanic respondents (*z*-score = −3.104, *p* < .001), and Native American and other race respondents (*z*-score = −1.964, *p* = .049).

Finally, a supplemental analysis in Appendix B assesses the relationship between each individual ACE and dental care utilization. These results show a negative and statistically significant relationship with dental care utilization among the following ACEs: parents separated (OR = .817, 95% CI = .718, .931), sexual abuse (OR = .793, 95% CI = .689, .912), physical abuse (OR = .759, 95% CI = .644, .895), emotional neglect (OR = .614, 95% CI = .614, .864), and physical neglect (OR = .728, 95% CI = .566, .937).

## Discussion

The current study found that women with four or more ACEs exhibited approximately 25% lower odds of dental care utilization during pregnancy than those without ACEs after adjusting for sociodemographic variables. This finding aligns with prior literature that revealed a link between accumulating ACEs and reduced dental care utilization among children and adolescents,^14–18^ and a smaller literature among adults in the United States.^19,20^ While the exact mechanisms of this relationship cannot be explored in the study it is possible that those exposed to ACEs may avoid dental care utilization due to patterns formed earlier in life, considering the link between ACEs and dental care in childhood and adolescence. In addition, those with more ACEs exposure may choose not to pursue dental attention due to apprehensions about reliving the trauma during a visit. Moreover, the findings also contribute to the existing literature by detailing that the relationship between ACEs and dental care utilization was most strongly concentrated among White and Native Americans. However, because the study used from two states with a large population of White and Native American persons relative to the United States,^34^ it is unclear whether this finding would generalize to a broader population.

This study results point to potential avenues for improving oral health and dental care access among women who experience ACEs during pregnancy. One possibility is greater coordination between dental care professionals and other health care providers, such as obstetrician-gynecologists counseling pregnant patients on the importance of oral health and dental care utilization during prenatal visits.^35^ Prenatal visits may be a critical window for interventions, including the referrals of information on local areas to acquire low-cost dental care services.^36^ Likewise, obstetrician-gynecologists could consider screening for ACEs at prenatal visits and provide additional guidance for pregnant patients, such as dental resources.^37^ Additionally, incorporating prenatal oral health guidelines and information during prenatal care visits may be beneficial.^35^

### Limitations and Future Directions

Certain limitations to the current analysis can be addressed in future research. First, it is important to note that our study only included data from North Dakota and South Dakota, given these states specifically asked questions about ACEs and dental care utilization during pregnancy. As a result, the generalizability of the results beyond these states may be limited, especially considering their unique characteristics, such as being more rural and having higher proportions of White and Native American individuals than the rest of the United States. Second, questions related to ACEs and dental care utilization may be susceptible to recall or social desirability bias, potentially influencing the accuracy of the responses. Third, the study focused only on dental care utilization during pregnancy because this was the only dental health-related question in the North and South Dakota PRAMS survey. However, it would be important for future research to consider other measures related to the relationship between ACEs and oral health issues during pregnancy, such as dental caries, periodontitis, or unmet dental care needs. In addition, it would be valuable for research to assess why individuals with more ACEs were less likely to use dental care services. Finally, due to the cross-sectional nature of the PRAMS data, it is important to interpret the findings as associations rather than indicative of causal relationships. Future research with longitudinal designs may provide a better understanding of causality.

## Conclusion

The current study provided valuable new information about the association between accumulating ACEs and dental care utilization during pregnancy. The results underscore the importance of further investigation to comprehend the underlying mechanisms that connect ACEs and dental care utilization during pregnancy. Additionally, there is a need to explore ways to provide trauma-informed support for individuals exposed to ACEs, aiming to improve dental care utilization during pregnancy.

## Figures and Tables

**Table 1:** Summary Statistics of Analytic Sample (*N* = 7,391)

Variable	Weighted Percentage
Dental Care Utilization	49.7%
*Number of ACEs*	
0	39.1 %
1	19.7%
2	11.1 %
3	8.1%
4+	22.0%

*Mother's Age*	
<18	0.8%
18–24	19.9%
25–29	34.7%
30–34	31.2%
35+	13.4%

*Mother's Race/Ethnicity*	
White	75.2%
Hispanic	5.2%
Black	3.9%
Native American	9.3%
Other Race	6.4%

*Mother's Educational Attainment*	
Less than High School	8.7%
High School Graduate	21.1 %
Some College	30.0%
College Graduate	40.2%

*Prior Births*	
0	34.7%
1	32.2%
2	18.1%
3+	15.1 %

Currently Married	68.4%
*Household Income*	
≤ $16,000	15.3%
$16,000, $40,000	18.1%
$40,001 - $85,000	36.3%
> $85,000	30.3%

*Number of Dependents*	
0	7.7%
1	31.1 %
2	29.0%
3	17.9%
4+	14.3%

Medicaid Recipient	14.0%


*Abbreviation:* ACEs = Adverse Childhood Experiences

**Table 2:** Logistic Regression of Dental Care Utilization During Pregnancy on Number of ACEs and Other Control Variables

	Full Sample (N = 7,391)	White (N = 3,589)	Hispanic (N = 710)	Black (N = 386)	Native American (N = 1,928)	Other Race (N = 778)
Number of ACEs	OR (95% CI)	OR (95% CI)	OR (95% CI)	OR (95% CI)	OR (95% CI)	OR (95% CI)
0 (Reference)	—	—	—	—	—	—
	—	—	—	—	—	—
1	1.037	1.064	1.425	0.745	0.730	0.902
	(0.881 – 1.220)	(0.872 – 1.298)	(0.749 – 2.712)	(0.365 – 1.518)	(0.513 – 1.039)	(0.491 – 1.656)
2	0.855	0.816	1.177	0.468	0.566**	2.055
	(0.698 – 1.047)	(0.639 – 1.042)	(0.545 – 2.543)	(0.150 – 1.463)	(0.374 – 0.857)	(0.923 – 4.577)
3	0.828	0.742*	0.725	1.865	0.683	1.743
	(0.653 – 1.049)	(0.556 – 0.990)	(0.315 – 1.666)	(0.526 – 6.608)	(0.452 – 1.032)	(0.740 – 4.107)
4 or more	0.745***	0.698**	1.691	0.885	0.558***	1.068
	(0.628 – 0.883)	(0.562 – 0.867)	(0.905 – 3.159)	(0.370 – 2.116)	(0.404 – 0.770)	(0.606 – 1.884)

*** p<0.001,

** p<0.01,

* p<0.05

*Note:* Control variables include maternal age, mother's race/ethnicity, mother's educational attainment, marital status, number of prior births, household income, Medicaid recipient, number of financial dependents, state of residence, and year of birth

## Data Availability

The data that support the findings of this study are available from the Centers for Disease Control and Prevnetion but restrictions apply to the availability of these data, which were used under license for the current study, and so are not publicly available. Data may be requested from the Centers for Disease Control and Prevention at: https://www.cdc.gov/prams/index.htm
